# Supplementary material for: Are carbon emissions trading and green financial instruments synergistic? -Comprehensive quantitative research based on content analysis
Source: PLoS One. 2024 Mar 7;19(3):e0298601. doi: 10.1371/journal.pone.0298601 (PMC10919601; doi:10.1371/journal.pone.0298601)
Supplement: S1 Data — (ZIP) [file pone.0298601.s001.zip › Data/地级市Z_F工作报告.zip!/╣π╢1⁄2/├╖╓▌╩╨/└┤╫╘╣1⁄2╓┌║┼í╛┬φ┐╦╩2╛▌═°í┐_2016.docx]

各位代表：

我代表梅州市人民政府向大会作报告，请予审议。请政协委员参议，请其他列席人员提出意见。

“十二五”主要成就及 2015 年工作回顾

“十二五”时期，市政府深入贯彻落实党的十八大和十八届三中、四中、五中全会精神，以及习近平总书记系列重要讲话精神，在省委、省政府和市委的正确领导下，在市人大和市政协的监督支持下，紧紧依靠全市人民，积极应对困难和挑战，致力改善发展条件，夯实发展基础，全力加快振兴发展步伐，经济社会发展取得明显成就，较好地完成了“十二五”各项工作任务。

——综合实力再上台阶。“十二五”末，全市生产总值达 955.09 亿元，比“十一五”末增长 63.7%，年均增长 10.4%；预计人均生产总值 22041 元，比“十一五”末增长 59.4%；一般公共预算收入突破 100 亿元，达到 103.58 亿元，是“十一五”末的2.66倍，年均增长 21.6%；8 个县（市、区）一般公共预算收入均超 5 亿元，其中梅县区在粤东西北 74 个县（市、区）排名第二；固定资产投资 568.06 亿元，是“十一五”末的 2.9 倍，年均增长 23.8%；完成进出口总额 24.54 亿美元，是“十一五”末的 2.09倍。

——产业结构明显优化。大力实施产业振兴行动计划，促进产业集约发展。三次产业比例由“十一五”末的 20.4：41.0：38.6 调整为 19.7：36.7：43.6，产业结构发生了积极变化。全市农业增加值年均增长 3.9%。五年新增 4 个省级工业园区，全市规模以上工业增加值年均增长 12.8%；先进制造业和高新技术工业增加值占规模以上工业增加值的比重，比“十一五”末提高 8.1 个百分点；烟草、电力、建材、电子信息、机电制造、矿产加工等六大产业工业增加值比“十一五”末增长 84.1%；电力、电子信息产业工业总产值均突破百亿元；技改投资年均增长 25.3%。第三产业蓬勃发展，实现增加值 416.54 亿元，比“十一五”末增长 65%。文化旅游产业加速发展，五年新增 1 个 5A 级、6 个 4A 级旅游景区和 22 家四星标准以上旅游酒店；信息产业方兴未艾，建成梅州家乐电商、梅县区电商等 10 家电子商务产业园，入园企业 320 家。金融对地方经济的支持力度加大，金融机构存贷比 47.1%，比“十一五”末提高 7.62个百分点。上市公司数量居粤东西北地区前列。城市经济加快发展，万达、碧桂园、富力、奥园等著名企业进驻梅州，五年投入 471.9 亿元建设客都汇、东汇城、万象江山、锦绣国际、海吉星等一批城市综合体和物流园。深入开展质量强市，完成首届市政府质量奖评选，建成省质量监督稀土产品检验站和深加工农产品检验站；五年全市新增省名牌产品 89 件，中国驰名、省著名商标 28 件，专利授权量 7221 件，大埔青花瓷获国家地理标志证明商标。全面完成省下达的节能减排任务，累计淘汰落后产能 1072 万吨，单位 GDP 能耗年均下降率为 3.89%，成功入选国家节能减排财政政策综合示范城市。

——交通环境显著改善。围绕建设“潮汕平原北上开拓腹地的交通枢纽”，以“两高一场”为重点，努力构建内外畅通的交通体系。“十二五”完成交通建设投资 317亿元，是“十一五”总投资的 2.6 倍。梅汕客专动工建设；新增 2 条出省高速通道，实现“县县通高速”目标；高速公路通车里程新增 223 公里、达到482 公里。梅县机场完成扩建，开通 9 条国际国内航线，年旅客吞吐量达 22.7 万人次。升级改造国省道 285 公里、县乡公路 1300 公里，完成新农村公路硬底化改造 3200 公里，具备通车条件的行政村客运通达率 100%。全面完成梅江、汀江、韩江航道整治工程。

——城乡面貌变化巨大。五年来，共投入城乡基础设施建设 537.9 亿元，年均增长 14%。梅县撤县改区，规划建设嘉应新区。中心城区建成区面积从 45 平方里扩大到 53.64 平方公里、人口突破 50 万。成功创建国家卫生城市并顺利通过复审，获评“中国十佳优质生活城市”。成为首批国家生态文明先行示范区。水利惠民工程成效显著，五年共完成城乡堤围除险加固 112.6 公里、病险水库除险加固 154 座、中小河流治理 92 宗，城区防洪标准达到百年一遇；扩建、改建 3 座城区自来水厂，建成 15座城区和中心镇生活污水处理厂。实施村村通自来水工程，解决 85 万农村人口饮水安全问题；农村“老旧”电网改造全面完成。美丽乡村建设有效推进，加强了镇村规划，完成 10 个历史文化名镇名村保护规划编制；40 个村列入中国传统村落名录，数量居全省首位；全市 17 个幸福村居示范片（线）加快建设。城乡环境得到有效整治，城区生活垃圾无害化处理率达 100%、农村生活垃圾有效处理率达 75.4%。

——农业农村工作取得新成效。“十二五”期间，完成高标准基本农田建设 42.93万亩，粮食生产保持稳定。金柚、茶叶等大宗经济作物种植面积扩大、效益提升。新型农业经营主体不断壮大，新增省级农业（林业）龙头企业 107 家；新增农民专业合作社 3176 家、家庭农场 852 家，组建了 12 个市级农业协会。依托客天下农电商产业园等平台，拓宽农产品销售渠道。我市入选全省第一批农业信息化示范市，梅县区被认定为“国家现代农业示范区”，平远县获评全国“电子商务进农村综合示范县”。农产品质量水平不断提高，新增 3 个国家地理标志保护产品，4 个农产品被评为广东“名米、名茶、名果”。农村综合改革不断深，蕉岭成为全省首个完成农村土地确权登记工作的县，全市 69 个镇铺开了农村土地确权工作。农村普惠金融试点、集体林权制度改革走在全省前列，设立 3 家村镇银行，2 个县农信社完成农商行改制工作，乡村金融服务站实现行政村全覆盖。成立全省首个林业产权流转服务平台。建成覆盖县、镇、村的农村“三资”管理服务平台，建立村民理事会试点镇 39 个、村民理事会776 个，农村基层治理水平不断提高。两轮扶贫开发“双到”任务全面完成，全市 937个省定贫困村和 8.147 万户、32.48 万贫困人口实现脱贫。

——群众生活水平持续提高。“十二五”期间，各级财政用于民生支出累计达913.57 亿元，年均增长 27.9%。城镇居民、农村居民人均可支配收入分别达到22029元和 11799元，年均增长 10.8%和 12.2%；城镇居民、农村居民人均生活消费性支出分别比上年增长 9.8%和 12.7%。城乡住户储蓄存款余额达 1061.2 亿元，比 2010 年增长 82.9%。每百户拥有小车 14.68 辆，是“十一五”末的 2.78 倍。创建“宽带中国”示范城市，宽带网络、有线电视网络实现行政村全覆盖。社会保险制度实现城乡全覆盖，“五大险种”综合参保人数达 833.16 万人次，比“十一五”末增长 34.4%。连续四年获评“中国最具幸福感城市”。

——社会事业加快发展。成功创建粤东西北地区首个教育强市，率先实现教育强县、强镇和“全国义务教育发展基本均衡县”全覆盖；义务教育阶段公办学校标准化学校覆盖率达 100%；支持嘉应学院实施“创新强校”工程，推动内涵发展、特色办学。优化配置医疗卫生资源，全市县级公立医院改革试点实现全覆盖，中山大学附属第三医院粤东医院正式运营。国家级客家文化（梅州）生态保护区规划启动实施。新增 3处国家级、36 处省级、80 处市级文物保护单位和 10 个省级非物质文化遗产项目。成功举办三届世界客商大会、三届客家文化创意产品博览会和 2013 年中央电视台中秋晚会，创作了《梦幻客都》、《客魂·家风》等一批文艺精品。获评“中国楹联文化城市”和“中华诗词之市”。联合国教科文组织设立的“印度洋之路”移民纪念项目在松口落成。梅县雁洋成为国内第二个“际慢城”，蕉岭获评“世界长寿乡”，梅县、大埔、丰顺获评“中国长寿之乡”。振兴足球之乡十年规划工作取得阶段性成效，我市被授予“中国体育非物质文化遗产保护与推广城市”；梅州五华队以中乙联赛第一名成绩成功“冲甲”，成为全国首支比赛主场设在县城的中甲足球队。完成 118 个农村文化俱乐部和 106 个乡镇农民健身工程建设。五年来，办成了一大批民生实事，底线民生标准持续提升；城镇新增就业 15.94 万人、劳动力转移就业 43.8 万人、培训劳动力 19.9 万人，扶持创业 1.8 万人，城镇登记失业率保持在 2.45%左右；新增养老机构 40 间、养老床位 19035 张，分别是“十一五”末的 1.3 倍和 11.6 倍；实现 80岁以上高龄老人补（津）贴全覆盖，为 80 岁以上老人、60 周岁以上困难群体购买意外伤害保险；给 1 万名重性精神疾病患者免费发放药物，免费收治 500 名贫困重症精神疾病患者；梅州城区 60 周岁以上老人和小学生、初中生等群体免费乘坐城区公交车，开通梅县区城市公交；完善全市治安视频网、社区群防群治网建设；投资 54 亿元，开工建设各类保障性住房 2.91 万套，棚户区改造安置房 20241 户（套）。创新实施“客都民意警务”，五年均获评全省综治工作优秀市，群众安全感和对政法工作满意度多年位居全省第一。全市信访总量逐年下降，积案存量逐步减少。新增社会组织 810 家，发展 273 个志愿组织，志愿者登记注册突破 15 万人。“六五”普法任务全面完成，“一村（社区）一法律顾问”实现全覆盖。扎实开展“三打两建”专项行动，有效净化了市场和社会环境。我市成为广东唯一全域被确认为原中央苏区范围的地级市。成立广东雁洋公益基金会，在民生、教育、扶贫、社会保障等领域广泛开展公益慈善活动。落实安全生产“党政同责、一岗双责、失职追责”责任体系，建立健全各项监管机制，安全生产形势持续稳定。国防教育和动员、兵役、双拥等工作扎实推进，成功创建全国双拥模范城市。实施“单独两孩”政策，计划生育工作取得新成效。外事侨务、港澳和对台工作有新进步，民族宗教、人事编制、妇女儿童和食品药品监管、统计、物价、供销、气象、打私、应急、人防、档案、方志、残联等工作取得新成绩。

——重点领域改革深入推进。大力推进简政放权和职能转变，社会和市场活力进一步释放。开展行政审批制度改革，市级行政审批事项压减 53.7%，审批时限缩短64%。完成工商、质监管理体制调整工作。全面实施商事制度改革，全市各类市场主体两年新增 5.3 万户。完成市、县两级权责清单公布工作，实行“三单”管理。深化投融资体制改革，成立市公共资源交易中心。顺利推进全市公务用车制度改革。国企改革稳步推进。

——政府自身建设得到加强。健全科学决策、民主决策机制，修订完善市政府工作规则。坚持依法行政，自觉接受市人大及其常委会的监督和政协的民主监督，认真听取各民主党派、工商联、无党派人士及工青妇等人民团体意见。五年来，共办理省人大建议 14 件、省政协提案 20 件和市人大建议 283 件、市政协提案 1006 件，办复率和基本满意率均达 100%。行政复议案件受理率、结案率均达 100%。我市成为全省首批获得地方立法权的设区市之一。大力实施 252 项“微改革、微创新”项目，优化了行政流程，提升了行政效能。行政服务实体大厅、网上办事大厅和“12345”政府热线等服务平台建成运行，市直部门行政审批事项 100%进驻网上办事大厅，方便了群众和企业。开展“梅州发布”微访谈，畅通民意诉求表达渠道。“梅州发布”获评人民网“十大最具亲和力政务微博” 。2015 年政府公共服务总体满意度居全省第六位、粤东西北地区第一位。扎实开展党的群众路线教育实践活动，严格执行“八项规定”，积极践行“三严三实”，持之以恒纠正“四风”问题，全市“三公”经费支出同比降低 28.4%。建立市、县两级网上效能电子监察系统，强化重点领域审计监督。

2015 年是“十二五”收官之年，市政府坚持稳中求进，围绕稳增长、调结构、促改革、惠民生，落实“两大振兴政策”，聚焦“三大抓手”，大力推进“一区两带”建设，狠抓各项工作落实，为“十三五”良好开局奠定了较好基础。全市生产总值增长 8.6%，一般公共预算收入增长 21.5%，固定资产投资增长 39.4%，主要经济指标增速均居全省前列。

一年来，我们主要抓了以下几方面工作：

一是突出交通、水利等基础设施建设。充分发挥投资的重要作用，全市 235 项重点项目完成投资 556 亿元，增长 78.7%。梅汕客专建设进展顺利，龙川—梅州—龙岩高铁、鹰梅铁路、浦梅铁路等项目前期工作加快推进。济广高速平兴段、汕湛高速五华段建成通车，兴华高速公路全线动工，梅平、大潮（含大漳支线）高速公路开工建设。新机场预选场址初步确定。水利能源等重大基础设施建设有重大突破，梅州抽水蓄能电站、广东省高陂水利枢纽工程、塔牌年产 600 万吨水泥生产线和梅州卷烟厂技改工程等一批重大项目动工建设；全面启动山区中小河流治理，完成 511.9 公里年度治理任务；粤电大埔电厂首期一号机组建成投产220 千伏变电站实现县（市、区）全覆盖。

二是狠抓实体经济发展。大力推动产业园区扩能增效，规划建设广东梅兴华丰产业集聚带，并上升为省级发展平台。加快建设畲江至梅城、兴宁、五华县城等 3 条快速干线，保税物流、高铁商务区、空港经济区和创新创业孵化园、联邦快递等一批重点项目正在抓紧推进，医院、学校等公共服务配套设施同步推进，综合保税区、国家经济技术开发区申报工作正在有序进行。全市园区实现规模以上工业增加值 68.49 亿元，产业集聚带入园项目累计 480 个。落实扶持实体经济发展政策，建立市直机关干部挂点服务企业制度。新建投产规模以上工业企业 7 家，全市规模以上工业增加值220.89 亿元，增长 9%。大力发展电子商务、文化旅游、现代物流、现代金融等新经济业态，推动大众创业、万众创新，为经济发展注入了新的活力。全市社会消费品零售总额 555.5 亿元，增长 11.1%。规划建设梅江韩江绿色健康文化旅游产业带，旅游总收入、旅游接待总人数分别增长 23%、20.1%。发展和利用资本市场成效明显，新增 2 家上市企业和 5 家“新三板”挂牌企业。金融机构存贷款余额分别达 1565.28 亿元和 736.41 亿元，分别增长 10.9%和 15.9%。

三是大力推进中心城区扩容提质。完成城市总体规划编制工作。统筹推进江南新城、芹洋半岛、梅县新城建设，调整优化江南新城规划，基本完成江南新城 6.66 平方公里征拆任务，安置区、市政道路、地下综合管廊、马鞍山公园和芹洋湿地公园等公共设施建设有序推进。注重产城融合发展，引进了一批优质产业项目，建成一批城市综合体。优化新区开发资本结构，成功争取粤东西北振兴发展股权基金和国家政策性银行贷款支持，利用国家棚户区改造政策，推行“PPP+政府购买服务”等建设模式，拓宽项目融资渠道。抓好中期土地规划调整，积极盘活存量建设用地。各县（市）新区建设扎实推进，梅县区雁洋镇、客天下旅游产业园列入省新型城镇化“2511”试点。

四是强力抓好招商引资和对外开放。以招商引项目聚产业，深入实施“乡贤回乡投资兴业工程”，突出产业招商、以商引商。完善招商引资激励机制，制定领导挂钩招商和联席会议制度。全市共引进合同项目 258 个、投资总额 913.84 亿元，其中亿元以上项目 196 个。发挥侨乡优势，以“汇聚客商力量、共创海丝未来”为主题，圆满举办第四届世界客商大会。大会突出经贸招商、项目建设和扩大开放，现场签约项目81 个、计划投资总额 362.44 亿元；在海外设立 14 个经贸文化联络处和 10 个旅游推广中心，与“一带一路”沿线国家和地区的文化、经贸、旅游交流合作进一步加强。

五是全面完成第二轮扶贫开发“双到”工作。借力省直相关单位和广州市对口帮扶，坚持“靶向疗法”，重点实施产业扶贫、学技扶贫，增强自我发展能力。筹集“双到”资金 27.05 亿元，实施了 12418 个产业、就业、助学及基础设施建设等帮扶项目。完成农村住房改造 38853 户，搬迁“两不具备”村庄 7665 户。贫困村集体经济收入平均达到 9.87 万元，贫困户年人均纯收入 8893 元，分别比帮扶前增长了 14.8 倍和2.36 倍。

六是致力保护生态、改善民生。深入开展绿满梅州大行动，全面完成“一消灭三改造”任务，完成碳汇造林 50.85 万亩，建成 120 公里高速公路沿线的生态景观林带；规划新建 34 个森林公园、5 个湿地公园；全市森林覆盖率达 74.78%，居全省第二位。建立重点环境问题市政府挂牌督办制度，加强跨县河流水质管理和考核，城区空气质量优良率达 96.1%，饮用水源地水质达标率 100%。国家环保模范城市、国家森林城市创建工作扎实推进。坚持民生为重，81.1%的财政支出用于民生建设，办好了十大民生实事。

各位代表，五年的砥砺奋进，开创了全市改革发展稳定的崭新局面，奠定了振兴发展的良好基础。在这里，我代表市政府，向全市人民，向中央和省属驻梅单位、驻梅部队，向所有关心支持梅州发展的海外侨胞、港澳台同胞和社会各界人士，表示衷心的感谢并致以崇高的敬意！

各位代表，回首五年来的历程，成绩令人鼓舞，经验弥足珍贵。我们深深体会到：必须从梅州的实际出发，扬长补短，不断完善发展思路，狠抓交通水利等基础设施建设，狠抓产业园区建设和发展实体经济，狠抓中心城区扩容提质，才能夯实梅州加快发展的基础。必须抢抓发展机遇，争取和利用好原中央苏区、粤东西北两大振兴政策，动员社会各方力量，聚焦产业和项目建设，才能增强发展动能，提升发展质量。必须改革创新、开放发展，以改革破解难题、以创新推动发展、以开放汇聚能量，才能激发市场和社会活力，推动梅州经济行稳致远，社会稳定和谐。必须务实为民，始终站在群众的立场想问题、办实事、解难题，让广大群众有更多的获得感和幸福感，才能汇聚发展的最大力量，实现富庶美丽和谐幸福梅州的梦想。

在看到成绩的同时，我们也清醒地认识到，我市经济总量小、人均水平低，与振兴发展目标还有较大差距。一是产业层次低，工业短板突出，重大项目和优势骨干企业少，支撑振兴发展的根基尚不牢固。二是“外通内连”的综合快速交通网络尚未形成，生产要素集聚度低，物流和生产成本较高。三是中心城区产业积聚度低，未能形成产城互促，对全市城乡的辐射带动能力弱。四是城乡基础设施欠账多，基本公共服务供给能力弱，与人民群众的需求还有较大差距。五是少数政府职能部门依法履职不到位，依法行政水平和服务群众的能力还有待进一步提高，等等。对此，我们将高度重视，采取切实有效措施认真加以解决。

“十三五”主要目标任务和 2016 年工作安排

各位代表，“十三五”时期是梅州全面建成小康社会的决胜阶段。市委全会审议通过的《关于制定梅州市国民经济和社会发展第十三个五年规划的建议》，确定了我市“十三五”时期经济社会发展的指导思想、基本原则、目标要求和主要任务。根据市委《建议》，市政府编制了“十三五”规划纲要（草案），提请本次大会审议通过后实施。

《纲要》提出，到 2020 年，全市经济社会持续健康发展，人民生活水平显著提高，生态文明建设取得新突破，全面深化改革和全面依法治市扎实推进，全面建成小康社会，为实现振兴发展目标打下坚实基础。全市 GDP 力争突破 1500 亿元，年均增长 9%左右；人均 GDP 年均增长 8%左右；城镇居民和农村居民人均可支配收入年均分别增长 9%和 10%；三次产业结构进一步优化；嘉应新区起步区基本建成扩容提质示范区，带动各县（市、区）新区全面开发；广东梅兴华丰产业集聚带建设成为广东山区重要的绿色产业发展基地和区域经济合作的新平台；梅江韩江绿色健康文化旅游产业带打造成为国家养生休闲产业创新区和全区域休闲度假目的地。围绕实现上述目标，

《纲要》还提出了“十三五”时期经济社会发展的工作任务和措施。

在今后工作中，我们将深入学习贯彻习近平总书记系列重要讲话精神，坚持用讲话精神统一思想、武装头脑、指导行动。牢固树立并切实贯彻创新、协调、绿色、开放、共享的发展理念，坚持人民主体、科学发展、改革创新、扩大开放、依法治市和党的领导六大原则，大力实施“一区两带”发展战略，破解发展难题，厚植发展优势，提升发展的整体性。一是以创新发展为动力，深化体制机制改革，加强科技、企业和产业创新能力建设。着力完善“一区两带”产业布局，加快发展电子信息、生物医药、文化创意、健康养生、新能源汽车等新型产业，改造提升烟草、电力、建材、五金机电、陶瓷等传统产业，扎实推进农业现代化，努力提升信息化建设水平，推动产业向中高端迈进，提高产业积聚能力，提升经济社会发展的质量和效益。二是以协调发展为导向，坚持统筹兼顾，着力解决交通基础设施、公共配套服务、县域经济、社会事业等发展不平衡问题。突出抓好交通能源水利等基础设施建设，强化中心城区和县城的核心带动能力，建设一批经济强县和中心镇，推动文化教育等各项事业全面进步，努力构建城乡共同发展、区域共同富裕，物质文明和精神文明更加协调、社会与经济发展更加和谐的新格局。三是以绿色发展为保障，把绿色发展理念融入社会发展各方面。按照主体功能区布局，建设国家生态文明先行示范区，推进生态修复和生态建设，着力构建绿色、低碳、循环产业体系，全面促进资源节约高效利用。积极倡导绿色生产方式、生活方式和消费模式，加强环境保护和污染治理，建设天蓝、地绿、水净的美好家园，增强发展的可持续性。四是以开放发展为牵引，坚定不移扩大对外开放。积极参与“一带一路”建设，加强与东南亚沿线国家和珠三角、汕潮揭、海西区、赣苏浙、港澳台等地的交流合作，致力打造优质营商环境，鼓励、吸引乡贤回乡投资兴业，推进外经贸发展转型升级，大力发展开放型经济。五是以共享发展为根本，建立完善民生保障各项体制机制。致力推动城乡基本公共服务均等化，大力推进大众创业、万众创新，提高就业创业和收入水平，实施精准扶贫、源头治贫，打赢脱贫攻坚战。积极创建卫生强市，打造健康梅州，努力保障人民健康。切实维护社会和谐稳定，让全市人民共建共享发展。

2016 年是“十三五”规划的开局之年，也是本届政府任期的最后一年，做好今年工作意义重大。我们必须看到，全球经济仍延续疲弱复苏态势，不确定、不稳定因素比较多，经济和贸易增长呈现“双低”态势。从国内看，在“三期叠加”的大背景下，经济增速趋势性、周期性放缓，经济下行压力依然较大。与此同时，我们也要看到，国家经济长期向好的基本面没有改变，尤其是我国从供需两侧发力推进结构性改革，实施“去产能、去库存、去杠杆、降成本、补短板”五大措施，供给体系质量和效率、有效投资将得到提高，经济增长内涵将发生深刻变化，经济发展方式加快转变，发展的活力将全面迸发；省委、省政府在大力推动粤东西振兴发展的同时，对原中央苏区和革命老区采取更加特殊的扶持政策，将为我市振兴发展提供更强的动能。从梅州自身情况看，这几年通过大力推进“三大抓手”工作，聚焦产业和项目建设，打基础、建平台、聚产业，加快发展的基础更加扎实，加上我们有“两大振兴政策”的强力支持，以及广州市的全面对口帮扶，可以说未来的发展潜力巨大。只要全市上下凝心聚力、群策群力，既保持忧患意识，做好应对更大困难挑战的准备，又对经济长期向好充满信心，扎实抓好当前各项工作，全市经济社会一定会保持良好的发展势头。

今年政府工作的总体要求：深入贯彻落实党的十八届五中全会、中央经济工作会议、习近平总书记系列重要讲话精神，按照省委、省政府和市委的决策部署，以“创新、协调、绿色、开放、共享”五大发展理念为引领，扭紧“三大抓手”，深入实施“一区两带”发展战略，着力推进交通等基础设施建设，着力发展实体经济，着力推进新型城镇化，着力发展社会事业，着力改善民生，确保“十三五”经济社会发展取得良好开局。

综合考虑各方面因素，今年全市经济社会发展的主要预期目标：生产总值增长 9%左右，固定资产投资增长 30%左右，社会消费品零售总额增长 12%，进出口总额增长 5%；一般公共预算收入增长 10%以上；居民消费价格指数控制在 3.5%以内；居民人均可支配收入增速与经济增长基本同步，城镇登记失业率控制在 3.5%以内；节能减排降碳约束性指标完成省下达年度计划。

实现今年的目标任务，重点是抓好七方面工作：

一、紧扭 “三大抓手”，全力推进“一区两带”建设

着力推进交通等基础设施建设。致力打造潮汕平原北上开拓腹地的交通枢纽，加快构建对外综合快速交通体系，提升互通互联水平。加快推进兴华、梅平、大潮（含大漳支线）高速公路建设，确保无障碍施工；动工建设梅州东环高速、大丰华高速丰顺至五华段、兴汕高速五华至陆河段。确保梅汕客专实现无障碍施工，力争年底前动工建设龙川—梅州—龙岩高铁，继续抓好鹰梅、浦梅铁路前期跟进工作。加快推动梅县机场迁建。优化路网结构，升级改造国省道 120 公里，提高普通公路的技术等级和服务水平。完善交通运输配套设施，加快推进市中心枢纽汽车站建设，建成一批纯电动公交车充电站。抓好广东省高陂水利枢纽工程、梅州抽水蓄能电站建设，推动大埔电厂二期和华润热电联供等能源重点项目的立项建设工作。加强航道养护管理，保障航道安全畅通。

加快推进中心城区建设。实施梅州市城市总体规划（2015-2030），完成嘉应新区综合管线、市政道路、消防等专项规划编制，着力构建“多规合一”的城乡规划体系。以嘉应新区起步区建设为重点，按照国家地下综合管廊和海绵城市试点标准，优先推进公共基础设施建设，年底基本完成江南新城、芹洋半岛、梅县新城的主要道路、地下管廊主体工程和安置区建设。抓好客都大桥、罗乐大桥建设，促进江南新城、芹洋半岛和梅县新城互联互通。建设剑英湖公园、梅花山公园、城市馆、青少年科技馆等城市公共设施，加快新区学校、医院的规划建设和升级改造。推进新区一批重点产业项目建设。优化城区路网，加强交通管理，启动建设环城路等一批城区道路，规划建设一批人行天桥、公共停车场；优化城区公交线路和加密发车班次。改善城区供水条件，建设梅县新城水厂（首期），加快城区老旧供水管网改造。改造提升江北老城区，推进市人民医院和文化体育公园片区的城市更新改造，整合嘉应学院、攀桂坊、客家文化创意园等资源，打造人文秀区新亮点。建立租购并举的住房制度，发展住房租赁市场，鼓励房地产商适当降低房价，积极化解库存。理顺城市执法体制，推广应用数字化城管系统，提升城市管理精细化、常态化和长效化水平。

集中力量推进广东梅兴华丰产业集聚带建设。抓紧制定产业带总体规划实施方案。优先推进公共交通、环保设施和学校、医院建设，加快推进梅畲快线等 3 条快速公路，动工建设创新创业孵化园，抓好综合保税区、高铁商务区、空港经济区和联邦快递等一批重点项目建设。发挥广梅产业园的核心带动作用，加快园区扩能增效，全面推进申报国家级经济技术开发区工作。启动新一轮用地报批和征地工作，年底前完成园区安置区建设。大力支持各县（市、区）产业园区建设，推动基础设施配套和产业集聚取得新进展。

大力推进梅江韩江绿色健康文化旅游产业带建设。坚持高起点规划，基础设施先行。着重从供给侧发力，提升旅游产品的整体质量。顺应旅游和休闲度假产业市场的变化，引进有实力的专业团队，系统梳理梅江韩江两岸的生态、健康、文化、旅游等核心资源，增强梅州文化旅游和健康养生产业的核心竞争力。加强景区道路、停车场、旅游厕所、游客信息服务和供水供电、应急救援等基础设施建设，提升旅游业服务能力和水平。依托“一带一路”旅游联盟、原中央苏区红色旅游联盟等平台，广泛开展旅游合作，培育壮大旅游消费市场。充分利用微博、微信等新媒体，开展宣传推介，扩大旅游品牌知名度和影响力。加强与高等院校和企业合作，强化导游等从业人员培训，开展旅游志愿公益服务。实施“旅游+”战略，推动旅游产业与工业、农业、体育等深度融合，延长产业链条。

集聚要素抓实项目建设。认真研究、主动对接中央和省扶持苏区发展政策，加强项目谋划，有针对性做好项目启动的前期准备工作，争取更大政策支持。加强重点项目库管理，落实政府投资三年滚动计划，力争更多项目纳入国家和省项目“笼子”。充分利用“三旧”改造、棚户区改造和低丘缓坡等政策，挖潜盘活存量建设用地和查处违法用地，优先保障“一区两带”重点项目用地需求。创新重点领域投融资机制，积极推广 PPP 模式，加强与政策性银行合作，提高重大项目直接融资比重。

二、坚持创新发展，大力提升产业发展能力和水平

推进工业技术创新。落实省工业转型升级攻坚战三年行动计划，研究烟草、水泥、电力等支柱产业转型升级技术线路图，抓好企业项目建设、用工、技术改造和服务平台建设等环节，推动梅州卷烟厂、塔牌集团等骨干企业产品与技术升级。发挥省级检验检测平台作用，推动电声、稀土企业自主创新发展，加速形成产业集群。深化与高校和科研院所的合作，探索组建广东梅州新材料研究院。积极推进科技企业孵化器、中小企业公共服务平台建设，加大力度培育科技创新型企业。推进智能制造示范基地建设。加强质量强市工作，新增一批省著名商标和地理标志保护产品。

推进服务业态创新。积极推动“互联网+”行动计划，推动互联网和现代服务业加速融合，培育发展金融保险、跨境电商、文化创意、休闲度假、健康管理、服务外包、APP 等一批新业态。按创建国家电子商务示范城市的要求，做好各项基础性工作。培育一批生产服务型物流园和配送中心，重点推进广铁现代铁路物流园、深圳有信达保税物流等项目建设，促进快递业有序发展。加快引进商业银行及村镇银行，力促 4个县（市）纳入省农村普惠金融试点范围，年内完成 2 家以上农信社改制。支持各类文化创意产业园和文化旅游产业基地发展，致力在文化（包装）创意和互联网创业方面培育更多创新人才。

推进农业组织创新。加快转变农业发展方式，依托各类农业协会，培育壮大新型农业经营主体。巩固发展市场流通平台，依托海吉星农产品商贸物流园、毅德现代商贸物流城、广东华泰农兴农产品交易中心等平台，拓宽农产品线上线下销售渠道。建立新型农业社会化服务体系，培育发展一批有效的新型社会化服务组织，为农民提供全链条、专业化的生产经营服务。健全农业科技创新体系，强化产学研合作，加快推进市农科创新中心、台湾农民创业园、绿色生态水产创新示范园建设，提高农业产业化水平。推动农业开发与旅游健康养生产业融合发展，加快梅县锦绣国际茶博城、广东明珠养生山城、大埔茶文化旅游产业园、蕉岭奥园跨境商品展示中心等项目建设。培育发展富硒等长寿食品品牌农业。

推进创业创新。大力推动大众创业、万众创新，充分发挥梅州市创业创新投资引导基金、促进创业小额担保贷款基金和大众创业万众创新专项扶持资金的功能，引导社会资本进入创业创新投资领域，年底前实现创业孵化基地或众创空间在各县（市、区）全覆盖。积极兑现创业创新相关政策，支持企业和社会建立各类创业平台。落实省减税降费等政策措施，加大政府性收费清理力度，减轻企业负担。深入推进政府部门“微改革、微创新”，进一步优化行政流程、提高办事效率，促进创业创新活动有效开展。

三、坚持协调发展，努力提升发展的整体效能

提高县域经济综合实力。用好县域经济扶持政策，加大对县域基础较好产业的扶持，重点支持梅江区发展电子信息产业，梅县区发展机械装备制造和高端电子信息产业，兴宁市发展机电制造和食品加工产业，平远县发展稀土新材料、机械电子和建材家具产业，蕉岭县发展新型建材和长寿产业，大埔县发展电力和陶瓷产业，丰顺县发展电声产业，五华县发展五金机电、酒业药业和先进装备制造业，促进特色产业集约化、多元化发展，致力打造一批百亿产业和企业。联动发展镇域特色产业，每个县（市、区）抓好 2-3 个工贸、旅游或特色农产品专业镇。积极发展资本市场，促进具备条件的企业上市或在“新三板”挂牌。

统筹城乡发展。推进县域城镇体系规划建设，优化城镇总体空间布局，构建“县城—中心镇—镇”三级城镇体系。加快推进各县（市）新区建设，提升县城辐射带动能力。大力改善县城和乡镇公共服务供给能力，推动县镇医联体一体化试点建设。完善户口登记制度，增强中小城镇对农村人口的吸引力，鼓励农村人口和外来人口向城镇有序转移。扎实推进城镇棚户区和农村危房改造，着力解决住房困难群体的住房难问题。抓好山区中小河流治理和农田水利示范县建设。全面提升农村电网保障水平。加快推进农村人居环境综合整治，探索推进农村生活垃圾分片处理，年底前建成 17个中心镇污水处理厂。推进省级名镇名村、新农村示范片和幸福村居示范片（线）创建工作，加强对传统村落、历史建筑的保护利用。深化农村综合改革，规范引导农村土地承包经营权有序流转。加快创建国家农村金融改革创新综合试验区和农村信用体系建设区。

力促教育卫生均衡发展。推进教育现代化先进市创建工作，全面实施争先“六大工程”。巩固全国义务教育基本均衡县工作，落实好全市相关中小学校与广州等珠三角地区优质学校的结对帮扶，促进教育教学水平提高。加大教师培训力度，努力培育一批名校长、名教师。提高城区中小学校和幼儿园教育资源的供给能力。支持嘉应学院特色办学，发展优势学科。支持民办教育、职业教育和社区教育发展。深化医药卫生体制改革，实施卫生强基工程，全面推进市县人民医院、中医院、妇幼保健机构以及乡镇卫生院、村卫生站四级网络建设和管理，提高基本医疗和基本公共卫生服务水平，积极创建卫生强市。实施“全面两孩”政策，实现生育政策平稳过渡。

发展文化体育等社会事业。大力推进客家文化（梅州）生态保护区建设，整合各类公共文体设施和服务资源，升级改造中国客家博物馆和梅州市华侨博物馆，建立市非物质文化遗产展示中心，提升文化软实力。鼓励社会力量参与公共文化建设，办好第四届客家文博会。做好梅县九龙嶂革命旧址、中共南方工委旧址等 359 处革命旧址遗址的保护利用工作。推进足球综合改革，鼓励开展各类足球赛事，支持中甲、中乙球队发展和五华足球文化公园建设。加强与广州体育学院等院校及相关机构合作，培育发展休闲体育产业。深入开展全民健身活动。加强国防动员工作，支持军分区和人武部正规化、信息化建设。扎实做好国家安全、人防、双拥、对台、气象、防灾减灾、民族宗教、统计、方志、档案、打私、打假、应急等各项工作。

创新社会治理。挖掘客家“和文化”功能，完善民事民治管理机制。总结推广蕉岭村民理事会的治理经验，积极探索农村基层治理新路径。加大预防和化解涉农社会矛盾工作力度，推进镇、村（居）人民调解委员会规范化建设，培育一批骨干型社会组织，提升基层社会治理水平。着力构建立体化治安防控网络，继续实施“客都民意警务”，促进社会治安综合治理。加大力度抓好禁毒戒毒工作。深入推进“一村（社区）一法律顾问”工作。严格落实安全生产责任制和挂牌督办制度。加快推进基层食品监管技术支撑体系建设，提高食品药品安全风险防控能力。

四、坚持绿色发展，建设美丽家园

筑牢生态安全屏障。扎实推进国家生态文明先行示范区建设。实施山水林田湖生态保护和修复工程，维护生态系统完整性和生物多样性。争取大埔、丰顺调整纳入国家重点生态功能区，支持平远国家地质公园试点建设。积极创建国家森林城市，持续开展“绿满梅州”大行动，抓好林业重点生态工程建设，加强森林资源的抚育和管护，启动天然林资源保护工程。加强森林防火的责任追究，严厉打击各类破坏森林资源的违法行为。落实最严格水资源管理制度，深入开展南粤水更清行动，加强入河排污总量控制。开展重要水源保护、火源管控立法相关工作。探索建立生态环境损害责任终身追究制。

推动绿色低碳循环发展。坚持把绿色发展理念融入经济社会发展各个方面。推进森林碳汇开发，发展林下经济，促进林业转型升级。大力发展环保产业，培育壮大一批有潜力的环保企业。实施新能源汽车推广计划，推动云山汽车加快发展，提高电动车产业化水平。大力推广使用节能环保建材，提高建筑节能标准，加快发展绿色建筑。探索推进生活垃圾分类处置和再生资源回收。

严格城乡环境质量管理。加快创建国家环保模范城市，推进国家节能减排财政政策综合示范城市建设，扎实开展水污染、大气污染、土壤污染等重点整治。推进电机能效提升、清洁生产、园区循环化改造、节能技术改造等重点节能工程，完成黄标车、高污染燃料锅炉等淘汰任务，保持城区全年空气质量优良。完善市政府挂牌督办重点环境问题制度，推动环保地方立法。实施韩江上游跨省界流域污染治理等环保工程，推动规模化畜禽养殖污染集中治理。开展农村人居环境整治，力争农村生活垃圾有效处理率达 80%以上。

倡导绿色环保生活新风尚。加强绿色理念宣传引导，以群众喜闻乐见的形式，积极开展环保文化进社区、进企业、进学校、进农村活动，引导广大群众关注、参与、支持环保。大力开展节能减排全民行动，扎实推进节能型机关建设，动员企业建立环保志愿服务队伍，培养全民环保意识。倡导绿色低碳、文明健康的生活方式，鼓励全社会参与“公交出行、绿色生活”等环保活动，建设低碳家园，让爱护环境、绿色生活、享受生态成为社会新风尚。

五、坚持开放发展，激发经济增长的活力和动力

加强区域交流合作。擦亮松口“印度洋之路第一港”品牌，积极参与“一带一路”建设，发挥海外经贸文化联络处和旅游推广中心的作用，密切与沿线国家和地区的交流合作。构建“大客家”文化交流平台，扩大世界客都影响力。探索设立梅港青少年交流合作基金，建立梅港青少年交流基地。筹建蕉岭台商工业园，规划建设一批海峡两岸交流基地示范点。争取梅州列入赴台个人游试点城市。充分利用国内外两个市场、两种资源，认真实施率先基本实现粤港澳服务贸易自由化规划纲要和行动计划，完善口岸通关等平台，支持重点县、重点行业、重点企业开拓多元化市场，扩大进出口份额。加强与珠三角、海西区、赣南和汕潮揭等区域的经济互动，推进产业合作，构建对外开放新格局。

大力推进信息化建设。顺应信息时代的发展要求，争取提前建成“宽带中国”示范城市。加快 4G 网络建设，实现全市行政村、重点旅游景区、主干道路全覆盖。加快公共免费 WIFI 热点建设，扩大重点区域有效覆盖。抓好云呼叫中心建设，推动政府政务云发展。推进云计算 IDC 建设，创造条件建设广东省数据灾备重要基地。积极配合国家有关部委和省有关部门，在梅州城乡推进超高速无线局域网技术的应用和相关产业发展。推进信息技术创新，完善现代物流公共信息服务平台，加快构建信用、认证、标准、支付和现代物流体系，加强互联网安全治理，优化电子商务支撑环境。

努力扩大招商引资和招才引智。加强与在外乡贤的沟通联系，最大限度动员、鼓励和吸引在外乡贤回乡投资兴业。坚持产业谋划和产业招商并举，结合“一区两带”产业规划，制定重点产业发展指引和重大招商产业目录，采取有利于产业在梅州拓展的有效措施，吸引重大产业项目落户。借力广州对口帮扶，加强与省属、广州市属企业对接，力促梅兴华丰产业集聚带核心区的项目引进有重大突破。强化市县两级的招商责任，集中力量招引大项目、好项目，带动产业加快发展。实施高素质人才引进和企业家培养等重点人才项目，以优惠的政策和良好的环境，吸引更多的专家、各类技术人员、创新科研团队到梅州落户和施展才华。

六、坚持共享发展，增进人民福祉

强化社会保障。加大扩面征缴力度，实施全民参保计划，扩大社会保险覆盖面。提高城乡居民最低生活保障、农村五保供养等底线民生保障水平。完善救助管理服务体系。继续推进城乡居民大病保险工作，异地就医结算医院扩大到 40 家。实施就业优先战略，充分用好省扶持创业带动就业政策，重点扶持优秀创业项目，促进创业带动就业。

实施精准扶贫。扎实推进第三轮扶贫开发“双到”工作，大力实施“五个一批”工程，注重因地制宜、因户施策，精准滴灌、靶向治疗，重点在产业发展、基础设施建设、医疗、教育、金融等方面进行帮扶，探索多渠道、多元化的精准扶贫、精准脱贫新路径。落实机关企事业单位挂点责任，广泛动员全社会力量参与扶贫，全面完成年度帮扶任务。

关爱困难弱势群体。加大财政支持力度，加强对残疾人、农村留守老人、留守妇女、留守儿童、艾滋病人等群体的帮扶。建立健全困难弱势群体生活补贴制度，帮助解决实际困难。积极发展特殊教育，加强标准化特殊教育学校建设，推动城乡残疾学生全程享有教育、康复和保健服务。建立农村留守儿童台账，实施镇村动态管理。鼓励和支持社会力量兴办养老服务机构，提升社会养老服务水平。支持和发展志愿服务组织，开展形式多样的关爱活动，推动创建“志愿者之城”。

办好十件民生实事。一是保障底线民生。城乡居民基本养老保险基础养老金标准从每人每月 100 元提高到 110 元；城镇、农村低保补助补差水平分别从每月 375 元、173 元提高到 419 元、191 元；农村五保供养标准提高到所在县（市、区）上年度农村居民人均可支配收入的 60%以上；孤儿基本生活最低养育标准集中供养水平从每月1240 元提高到 1340 元、分散供养水平从每月 760 元提高到 820 元；城乡医疗救助人均补助标准从每年 1556 元提高到 2178 元，政策范围内住院医疗救助比例提高到70%以上。二是帮扶困难弱势群体。用于社会福利事业的彩票公益金，50%以上的资金用于支持发展养老服务业。为重度残疾人、精神和智力残疾人缴纳城乡居民养老保险费。建立事实无人抚养儿童生活津贴制度，标准为每人每月 500 元。新开工建设9024 套（户）城市棚户区改造安置房（含货币补偿）和 985 套（户）公共租赁住房（含租赁补贴），完成 7767 套（户）棚户区改造安置房和 518 套公共租赁住房建设。三是改善农村生产生活条件。完成新农村公路路面硬化改造 400 公里。完成 34 宗 64项 660.4 公里山区中小河流治理工程，完成 30 宗新增小型病险水库除险加固任务。将行政村全部纳入农村基层组织经费保障补助范围，提高村办公经费补助和村干部补贴。完成县、镇、村（社区）三级公共服务平台建设。支持农村光缆建设。四是提高城乡居民基本医疗保障水平。城乡居民医疗保险补助标准从年人均 380 元提高到 410 元。市内二级医院住院支付比例从 70%提高到 80%，市内三级医院住院支付比例从 55%提高到 65%，市外医院支付比例从 50%提高到 60%；基金年度累计最高支付限额从 20 万元提高到 45万元（不含大病保险）；将大病保险起付标准从 1.5 万元降为 1.2 万元，年度累计 6万元以下部分、报销比例从 50%提高到 60%，年度累计超过 6 万元（含）以上部分、报销比例从 60%提高到 70%，年度累计最高支付限额从 12 万元提高到 15 万元；提高符合计划生育政策分娩补助标准，顺产补助从 700 元提高到 1000 元、剖宫产补助从 1000 元提高到 1300 元。五是优化文化卫生体育及法律援助公共服务。基本公共卫生服务经费标准提高到每人每年 40 元以上。规划建设东山医院（嘉应学院医学院附属医院）和广州中医药大学紫合梅州医院，改造建设梅州市中医医院（田家炳医院）；建设市县两级出生缺陷综合干预中心，对孕产妇产前筛查和新生儿疾病筛查予以补助；35-64 岁城乡贫困妇女免费检查乳腺癌、宫颈癌。建设 100 个农村文化俱乐部；在梅州城区新建 20 个文化驿站（台）。建设 9 个社区体育公园；升级改造城区东较场游泳池、田径场和 3 个足球场。将法律援助的对象从低保群体拓展到低收入群体，将刑事法律应当援助扩大至侦查、起诉和审判三个阶段，对困难职工援助从工伤、欠薪扩展到家庭婚姻保障方面。六是促进教育资源公平。市属民办、县属中等职业学校免学费补助标准从每人 3000 元提高到 3500 元，市属公办中等职业学校免学费补助标准从每人3500 元提高到 4000 元。农村寄宿制学校生均住宿费补助标准从每年 150 元提高到200 元，学前教育困难家庭幼儿资助标准从每年 300 元提高到 1000 元。新建 4 所标准化特殊教育学校。将山区和农村边远地区学校教师补贴政策实施对象扩大到公办普通高中和公办幼儿园。规划建设梅州南方紫琳职业学院、梅江区风眠小学和芹洋学校，扩建梅江区伯聪中学和水白中学。七是扶持创业就业。劳动力技能培训补贴 1 万人，促进创业2500 人，带动就业 1 万人。向珠三角地区输送劳动力约 1 万人，城镇新增就业 3 万人，失业人员再就业 2.2 万人，就业困难人员再就业 3000 人。扶持 2000 名残疾人自主创业就业。八是加强生态建设和环境治理。规划新建 21 个森林公园、2 个湿地公园。建设 24 个市级乡村美化绿化示范点。规划建设梅兴华丰产业集聚带及梅江干流沿河镇的污水处理设施。新建 74 座、改扩建 49 座旅游厕所。完成 106 个集中式乡镇饮用水源保护区规范建设。定期进行生活饮用水水质检测和信息公布。九是实施网络信息惠民工程。启动“电信诈骗防火墙”工程。提速光纤网络宽带，新开通 875 个行政村光纤入户（住户）业务，城市及乡镇中心区域光纤宽带普及率达 85%；新增一批公共移动通信基站、基站站址和公共区域无线局域网络热点、无线 AP 点；新增快递末端服务网点100个，覆盖全市104个乡镇。推广应用互联网交通安全综合服务平台。建设 114 个标准化乡镇气象信息服务站。十是保障公共安全。规范出租屋管理，出租屋安装应用“门禁+视频”系统 5000 套。建成 10 个社区微型消防站。创建 1 个省级地震安全示范社区。实施 100 公里县乡公路安全生命防护工程。在全市食用农产品批发市场、农贸市场开展食品安全快速检测工作。

七、从严从实，建设为民务实清廉政府

深化行政管理改革。以转变政府职能为核心，提升服务经济发展能力。推进基本建设项目审批制度和流程改革，清理规范行政审批中介服务；深化商事制度改革，全面推进“三证合一、一照一码”改革，实行企业注册登记“同城通办”；严格执行“三单”管理制度，出台实施市政府投资管理办法，推进投资管理体制改革。落实供给侧改革各项政策措施，以创新供给带动需求拓展，提高企业产品质量和效益。实施机关事业单位养老保险制度改革，探索推动医疗保险城乡一体化改革。基本完成全市国有林场改革任务。大力推进电子政务，健全行政服务中心、网上办事大厅服务功能，继续开展“梅州发布”微访谈，擦亮“12345”政府服务品牌。完善领导干部、机关干部挂点联系基层和企业制度。

提高全面依法治市水平。自觉接受人大法律监督、工作监督和政协民主监督，主动听取民主党派、工商联、无党派人士意见建议，扎实办好人大议案、代表建议和政协提案。严格按照法定权限和程序行使权力、履行责任，全面推进政务公开。修订出台法治政府建设指标体系和依法行政考评办法，制订出台市政府规章制定办法，规范行政执法行为。成立市政府法律顾问室，推进行政复议委员会试点及“阳光复议工程”。开展“七五”普法工作。完善财政资金使用和管理办法，强化财政预算和专项资金审计。探索建立政府性债务风险预警体系，防范和化解财政风险。

加强公务员队伍建设。市政府党组带头深入学习习近平总书记系列重要讲话精神，把中央对党员领导干部的要求内化于心、外化于行。深化“三严三实”专题教育，深入开展“讲担当、比奉献”主题实践活动，激发公务员干事创业的干劲和热情。严格落实“三个区分”，鼓励担当，激励创新，整治为官不为、懒政怠政，凝聚改革创新正能量。严格执行中央八项规定和党风廉政建设责任制，落实《廉洁自律准则》、《党纪处分条例》，推进作风建设常态化、长效化。加强对重点领域、重点民生、重点工程的责任审计，有步骤推进审计全覆盖。开展谈话提醒、构建抓早抓小工作机制，筑牢防微杜渐的防线。

各位代表，站在新的起点，我们的事业任重道远。让我们紧密团结在以习近平同志为核心的党中央周围，以习近平总书记系列重要讲话精神为指南，在市委的直接领导下，抢抓机遇，戮力同心，聚众智谋发展，汇众力建小康，为梅州加快振兴发展，谱写美好生活新篇章而努力奋斗！
